# Homotypic and Heterotypic Protection and Risk of Reinfection Following Natural Norovirus Infection in a Highly Endemic Setting

**DOI:** 10.1093/cid/ciaa019

**Published:** 2020-01-09

**Authors:** Preeti Chhabra, Saba Rouhani, Hannah Browne, Pablo Peñataro Yori, Mery Siguas Salas, Maribel Paredes Olortegui, Lawrence H Moulton, Margaret N Kosek, Jan Vinjé

**Affiliations:** 1 Division of Viral Diseases, Centers for Disease Control and Prevention, Atlanta, Georgia, USA; 2 Bloomberg School of Public Health, Johns Hopkins University, Baltimore, Maryland, USA; 3 Oak Ridge Institute for Science and Education, Oak Ridge, Tennessee, USA; 4 Division of Infectious Diseases and International Health, University of Virginia, Charlottesville, Virginia, USA; 5 Investigaciones Biomédicas, AB PRISMA, Iquitos, Peru

**Keywords:** norovirus, homotypic protection, heterotypic protection, GII.4, reinfection

## Abstract

**Background:**

Norovirus is a leading cause of acute gastroenteritis worldwide, yet there is limited information on homotypic or heterotypic protection following natural infection to guide vaccine development.

**Methods:**

A total of 6020 stools collected from 299 Peruvian children between 2010 and 2014 were tested by norovirus real-time reverse-transcription polymerase chain reaction followed by sequence-based genotyping. Cox proportional hazards models were used to derive adjusted hazard ratios (HRs) of infection among children with vs without prior exposure.

**Results:**

Norovirus was detected in 1288 (21.3%) samples. GII.4 (26%), GII.6 (19%), and GI.3 (9%) viruses accounted for 54% of infections. Homotypic protection for GI.3 (HR, 0.35; *P* = .015), GI.7 (HR, 0.19; *P* = .022), GII.4 (HR, 0.39; *P* < .001), and GII.6 (HR, 0.52; *P* = .006) infections was observed. Hazard analysis showed that children with prior GII.4 infection exhibited heterotypic protection with a 48% reduction of subsequent GI.3 infection (HR, 0.52; *P* = .005). Prior exposure to GI.3, GII.2, and GII.17 infections enhanced susceptibility to subsequent infections with several other norovirus genotypes.

**Conclusions:**

Children up to 2 years of age infected with GII.4 noroviruses demonstrated both homotypic and heterotypic protection to reinfection with other genotypes. These data support the need for ongoing vaccine development efforts with GII.4 as the main component and caution the inclusion of genotypes that may enhance susceptibility to infections.

## Teaser

Infections with GII.4 noroviruses demonstrated both homotypic and heterotypic protection. However, increased hazard of subsequent infection following infection with several other common genotypes will require additional studies prior to the inclusion of additional genotypes in a multivalent vaccine.

**(See the Editorial Commentary by Atmar and Ramani on pages 230–2.)**

Noroviruses are associated with approximately 18% of all cases of acute gastroenteritis globally [1]. In children, noroviruses cause 70 000–200 000 deaths per year worldwide, of which the majority occur in developing countries [2]. Noroviruses are a genetically diverse group of viruses with a genome that consists of single-stranded positive-sense RNA of 7.5 kb in length. The genome of human norovirus is organized into 3 open reading frames (ORFs) of which ORF2 encodes the major capsid protein (VP1) [3]. Based on sequence differences of the VP1 protein, noroviruses are classified into 10 genogroups (GI–GX) and 49 genotypes with most of the infections in humans caused by GI and GII viruses, of which GII.4 viruses are the most prevalent genotype worldwide [4].

Although norovirus infects people of all ages, children are most susceptible to infections during their first years of life and they can, depending upon their environmental and health conditions, be reinfected multiple times with different norovirus genotypes [5–8]. Early human volunteer studies showed a lack of cross-protective immunity between viruses from the 2 major genogroups (GI and GII), and in 1 study volunteers did not develop clinical symptoms when rechallenged with the same strain 6 months after first challenge, suggesting that clinical immunity exists [7]. Recent insights suggest that the high titer of the challenge virus may not have mimicked natural exposure conditions [9, 10]. Birth cohort studies provide an excellent opportunity to follow natural infections under real-life conditions including duration of virus shedding [9]. In this study, we genotyped norovirus-positive samples from a longitudinal birth cohort in a highly endemic region in Peru [11] to quantify homotypic and heterotypic protection to norovirus genotypes naturally acquired during the first 2 years of life.

## MATERIALS AND METHODS

In 2009, the study of Etiology, Risk Factors, and Interactions of Enteric Infections and Malnutrition and the Consequences for Child Health (MAL-ED) was established in 8 countries that have a high incidence of diarrheal disease and malnutrition [12]. In Peru, the study site was located in 3 rural communities: Santa Clara de Nanay, Santo Tomas, and La Union [13]. From January 2010 to February 2014, 299 healthy newborns from communities in the Loreto province in Peru [13] were enrolled within the first 17 days of life and followed up to 24 months of age as a site of the MAL-ED study [12]. Enrollment was done using a well-defined recruitment protocol with stringent inclusion and exclusion criteria [11]. Mothers or caregivers of participating children provided written consent during enrollment and verbal consent at each follow-up visit. Stools were collected within 48 hours of a diarrheal episode, defined by a maternal report of 3 or more loose stools in 24 hours, or 1 loose stool with visible blood. Routine nondiarrheal stools were collected monthly from age 1 to 12 months and quarterly at 15, 18, 21, and 24 months.

A total of 6020 stool samples from 299 participants were collected of which 2192 samples were from diarrheal episodes and 3828 samples were routine nondiarrheal stools. All diarrheal samples and a 10% subset of routine nondiarrheal stool samples had previously been tested by TaqMan Array Card [14] as a component of the MAL-ED study. We tested the remaining nondiarrheal samples for norovirus by real-time reverse-transcription polymerase chain reaction (RT-PCR) (primers/probes: Cog1F, Cog1R, and ring1E probe for GI viruses; Cog2F, Cog2R, and ring 2 probe for GII viruses) and genotyped norovirus-positive samples (diarrheal and nondiarrheal cases) by a dual typing assay (MON432 and G1SKR for GI viruses; MON431 and G2SKR for GII viruses) followed by Sanger sequencing of the PCR products as described previously [15].

Viral nucleic acid was extracted on an automated KingFisher extractor (Thermo Fisher Scientific, Pittsburgh, Pennsylvania) from 10% clarified fecal suspensions prepared in phosphate-buffered saline using a MagMax-96 Viral RNA Isolation kit (Ambion, Foster City, California), according to the manufacturers’ instructions. Norovirus real-time RT-PCR was performed using the AgPath-ID 1-step RT-PCR kit (Applied Biosystems, Carlsbad, California) as published previously [15]. PCR products were visualized on a 2% agarose gel (Seakem-ME, Lonza, Allendale, New Jersey) containing Gel Red (Biotium, Fremont, California) and gel purified by an ExoSAP-IT (Affymetrix, Cleveland, Ohio) or QIAquick PCR purification kit (Qiagen) followed by Sanger sequencing (Eurofins MWG Operon, Louisville, Kentucky). Sequences were genotyped using online norovirus typing tools (https://www.rivm.nl/mpf/typingtool/norovirus/ (or https://norovirus.ng.philab.cdc.gov/) [16]. Genotypes were confirmed by phylogenetic analysis using norovirus reference sequence databases at the Centers for Disease Control and Prevention using MEGA X [17]. Maximum likelihood trees were computed with 1000 bootstrap replicates.

To generate estimates of overall prevalence and perform longitudinal analyses, we included all data from the MAL-ED Peru site in the analysis, resulting in an overall sample size of 6020 stools in statistical analyses from 299 distinct children. Survival analysis was conducted on data from 194 children (n = 4824 stools) who completed 2 years of surveillance. Kaplan-Meier survival analysis was conducted to estimate the time to the first GI/GII detection in samples from symptomatic and asymptomatic children. A Cox proportional hazards model was used to model the hazard of subsequent norovirus infections among children with vs without prior exposure for each genotype on the same genotype (homotypic protection) and all other genotypes (heterotypic protection) with >5% prevalence. Children entered the risk set at birth and were classified as unexposed until first recorded infection of interest, after which the time-varying exposure variable switched to “1” and they began contributing person-time to the “exposure” group. The model employed the Breslow method for ties and a robust variance estimate to account for within-child clustering at the level of the individual child, who contributed multiple observations to the risk set [18, 19]. The analysis was done allowing multiple failures per subject, and children were censored at the end of follow-up. We defined infections occurring within 30 days as markers of persistent infection and excluded them from analysis a priori based on current evidence and validated with sensitivity testing [20]. Final models were adjusted for duration of exclusive breastfeeding (days until first non–breast milk meal), and length-for-age *z* scores to represent nutritional status. An identical model was run for symptomatic GII.4 and GII.6 infections (the only genotypes with > 50 episodes of clinical episodes) to compare the protection generated from all infections vs that seen following symptomatic illness. We used the Holm method to correct for multiple comparisons. Recognizing that this is conservative, an exact binomial 1-sample test was also performed to determine if the number of the null hypothesis was likely to occur by chance alone and to compare this to the number of observed rejections for improved interpretation [21].

## RESULTS

**Figure 1.**
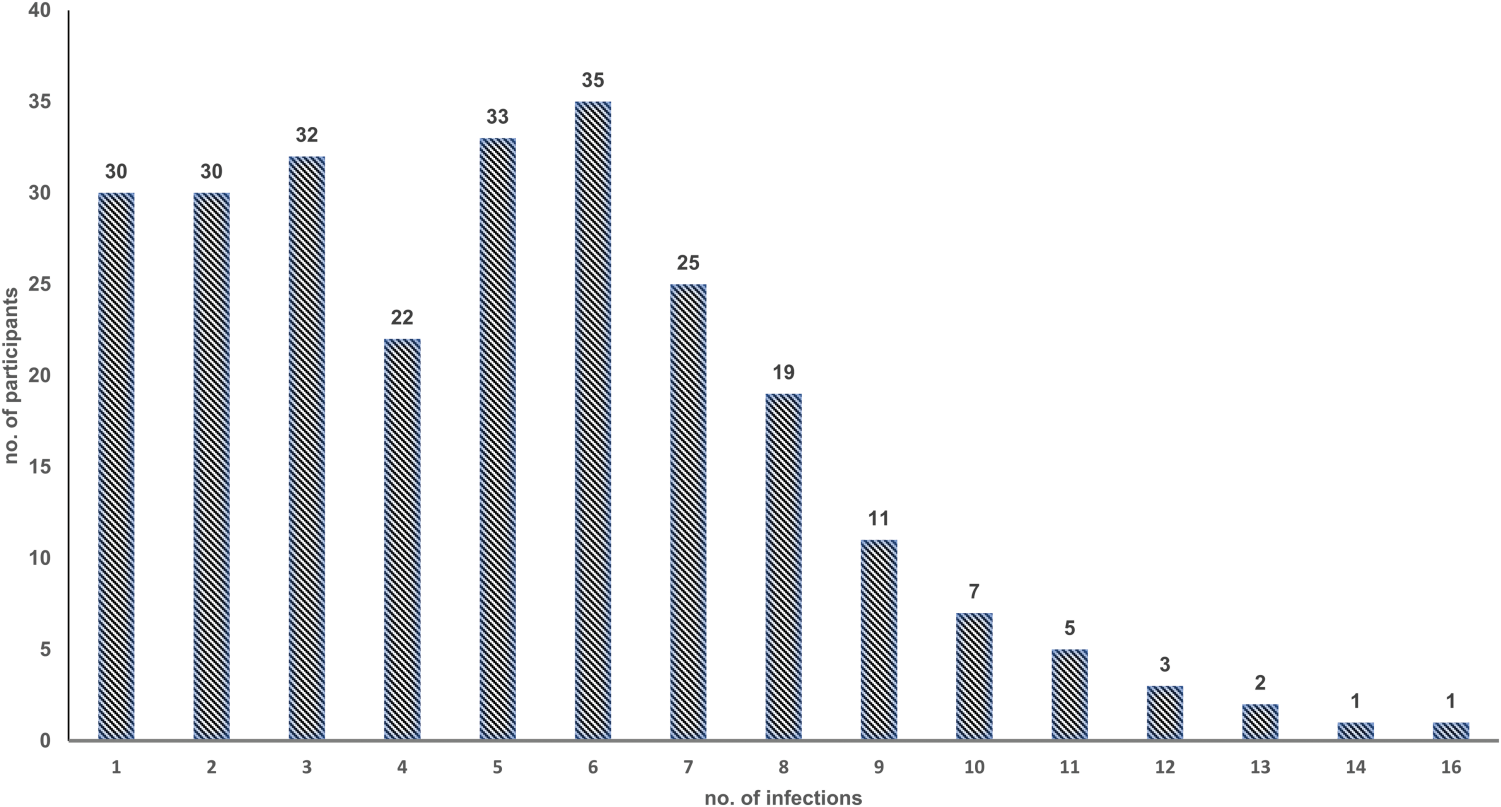
Number of norovirus infections per child in a Peruvian birth cohort during their first 2 years of life.

**Figure 2.**
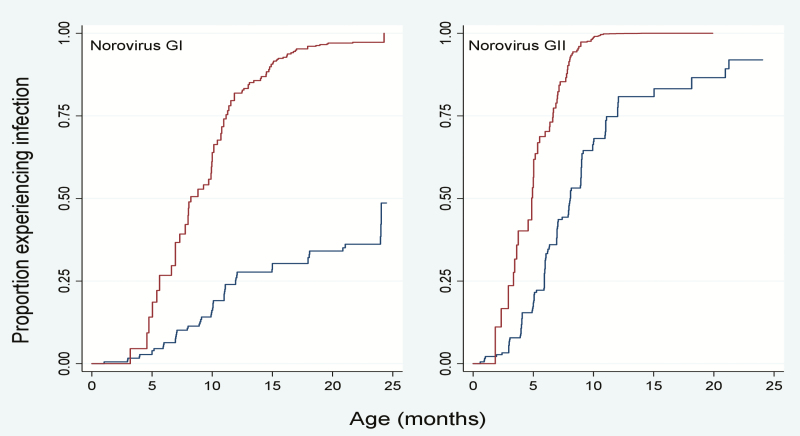
Kaplan-Meier survival analysis to estimate time to first GI and GII norovirus detection in symptomatic and asymptomatic children.

Overall, the prevalence of norovirus in diarrheal (n = 2192) and nondiarrheal (n = 3828) stool samples from 299 participants of the Peruvian birth cohort was 22% (n = 471) and 21% (n = 817), respectively. Two hundred fifty-six (86%) children had at least 1 norovirus infection throughout their 2 years of life and 109 (36%) children experienced >5 infections (Figure 1). Among norovirus-positive samples (n = 1288), 21.4% were positive for GI (n = 276), 73.7% (n = 949) were positive for GII, and 4.9% (n = 63) tested positive for both GI and GII (Table 1). In norovirus-positive diarrheal specimens, coinfections with *Campylobacter* and *Giardia* were the most commonly detected pathogens (Table 2). Among 194 children included in longitudinal analyses (n = 3001 nondiarrheal and 1823 diarrheal stool samples), the majority experienced a symptomatic norovirus infection prior to detection of the virus in routine nondiarrheal stools. The incidence of GI infections was low in children <5 months of age, rapidly increasing to 80% at 1 years of age. In contrast, symptomatic GII infections were detected in children as young as 2 months of age with nearly all children experiencing at least 1 symptomatic norovirus disease episode by their first birthday. At 2 years of age, asymptomatic infections were detected in 50% of children infected with GI viruses and 90% of the children with GII viruses (Figure 2).

**Table 1.** Prevalence and Distribution of Norovirus Infections in a Peruvian Birth Cohort, 2010–2014

Characteristic of Samples	Full Sample	Subsample for Longitudinal Analyses
Total No. of participants	299	194
Total No. of samples	6020	4824
No. of diarrheal samples	2192	1823
No. of nondiarrheal samples	3828	3001
Total No. of norovirus-positive samples	1288	1150
Total No. of norovirus-positive diarrheal samples	471	457
Total No. of nondiarrheal norovirus-positive samples	817	693
GI-positive samples, no./No. (%)	276/1288 (21.4)	250/1150 (21.7)
GII-positive samples, no./No. (%)	949/1288 (73.7)	839/1150 (73.0)
GI and GII–positive samples, no./No. (%)	63/1288 (4.9)	61/1150 (5.3)

**Table 2.** Frequency of Coinfection With Other Enteric Pathogens in Norovirus-Positive Samples From Symptomatic Children in the Peruvian Birth Cohort

Pathogen	No. (%) of Norovirus-Positive Diarrheal Samples Coinfected With Other Pathogens Detected	
	GI-Positive Samples (n = 116)	GII-Positive Samples (n = 390)
*Giardia*	38 (32.8)	101 (26.0)
*Astrovirus*	11 (9.5)	19 (4.9)
*Adenovirus*	6 (5.2)	13 (3.3)
*Campylobacter*	35 (30.2)	139 (35.7)
*Shigella*	4 (3.5)	7 (1.8)
EAEC	29 (26.9)	68 (18.3)
EPEC	4 (3.7)	14 (3.8)

Abbreviations: EAEC, enteroaggregative *Escherichia coli*; EPEC, enteropathogenic *Escherichia coli*.

**Figure 3.**
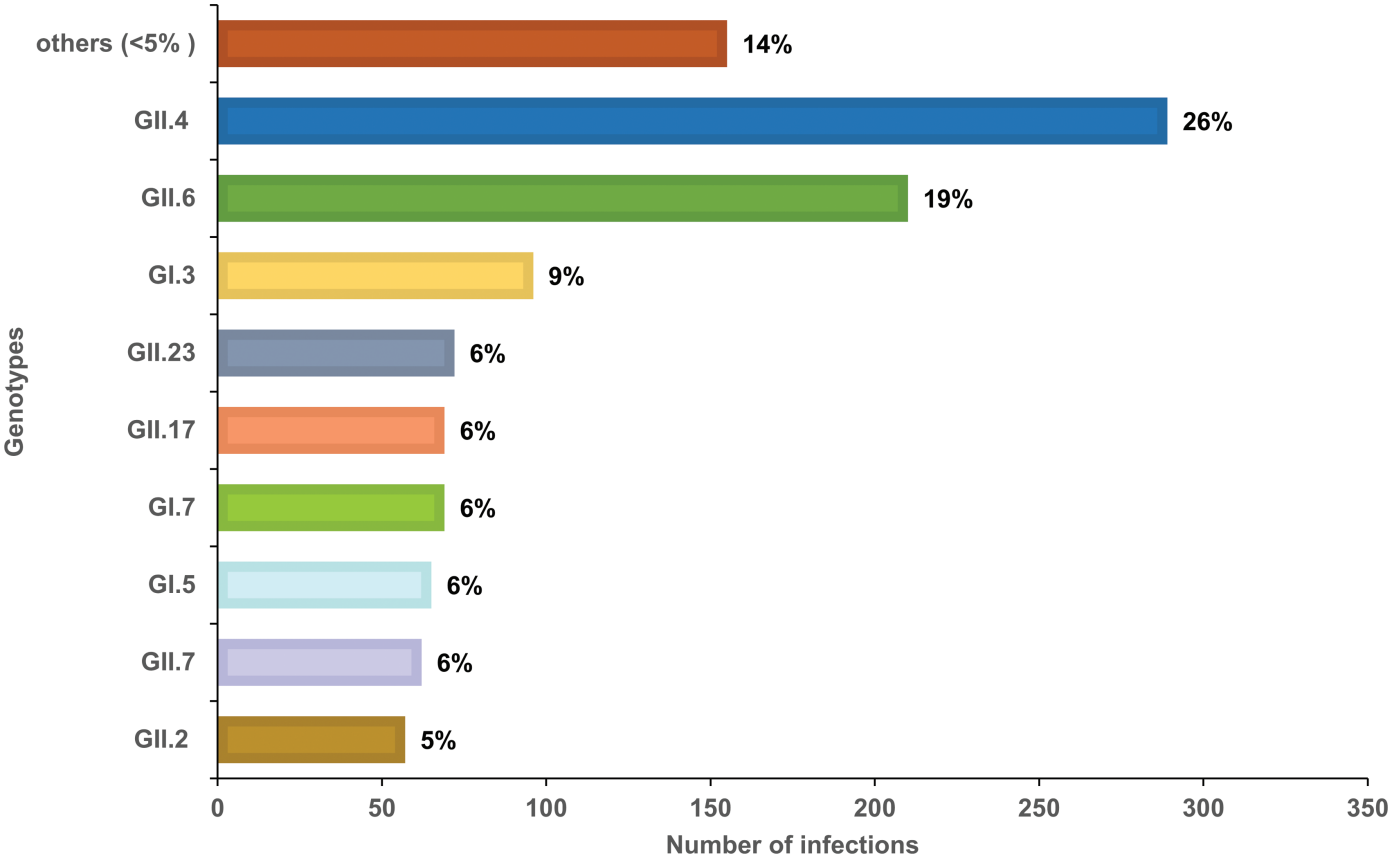
GI and GII norovirus genotypes in children from a Peruvian birth cohort from 2010 to 2014. Other genotypes include genotypes each with a prevalence < 5% (GI.1, GI.2, GI.4, GI.6, GI.8, GI.9, GII.1, GII.3, GII.8, GII.14, GII.22, GII.24, GII.26, GII.27, and GII.NA1 [a not-yet-assigned GII genotype]) [4].

A total of 1127 (87.5%) samples were genotyped successfully and the remaining 161 samples had cycle threshold values of > 30. All 9 established GI genotypes and 16 different GII genotypes were identified in this cohort, with GII.4 (26%) and GII.6 (16.1%) viruses as the most frequently circulating genotypes (Figure 3). The majority of the genotypes (84%) were associated with 1 P-type (eg, GII.6[P7], whereas GI.3, GII.4 New Orleans, and GII.17 viruses were detected with several different P-types (eg, GII.17[P31], GII.17[P13], and GII.17[P17]). Three recently reported novel genotypes (GII.23, GII.24, and GII.27) and 1 tentative novel genotype (GII.NA1) were also identified [4, 22].

A total of 989 norovirus-positive samples from genotypes that were detected at > 5% frequency (GI.3, GI.5, GI.7, GII.2, GII.4, GII.6, GII.7, GII.17, GII.23) were included in longitudinal analyses of homotypic and heterotypic protection. To distinguish reinfection from persistent infection, 151 samples that had been collected within 30 days from a prior infection with the same genotype were excluded from the analysis. Overall, 27 infections with the same genotype that are separated by 30 days or more occurred including 11 GII.4; 8 GII.6; 2 of each GI.5, GII.2, and GII.23; and 1 of each GI.3 and GI.7 infections. Prior infections with GII.4 and GII.6 were associated with homotypic reductions in risk, or reduced hazard of future infection with the same genotype by 61% and 48%, respectively (Table 3; HR, 0.39, *P* < .001 for GII.4; HR, 0.52, *P* = .006 for GII.6). Prior infections with GI.3 and GI.7 trended toward demonstrating homotypic immunity with decreased hazards of infection of 65% and 81% but failed to retain significance after correction for multiple comparisons (HR, 0.35, *P* = .015 for GI.3; HR, 0.19, *P* = .022 for GI.7). Significant evidence of heterotypic protection was observed among children with prior GII.4 infections, who exhibited a 48% reduction in the hazard of subsequent GI.3 infection (HR, 0.52; *P* = .005) and a trend of a 59% reduction in the hazard of subsequent GII.2 infections (HR, 0.41; *P* = .016) that failed to reach significance after correction for multiple comparisons.

**Table 3.** Adjusted Hazard Ratios Denoting Homotypic and Heterotypic Protection or Cross-reactivity Between Norovirus Genotypes Among 194 Children in Peru

Prior Infection	Adjusted Hazard Ratio (95% CI)							
	GI.3 (n = 73)	GI.5 (n = 51)	GI.7 (n = 58)	GII.2 (n = 45)	GII.4 (n = 195)	GII.6 (n = 150)	GII.17 (n = 53)	GII.23 (n = 49)
GI.3	**0.35** ^**a**^ **(.15–.82)**	0.64 (.28–1.48)	0.60 (.27–1.34)	0.89 (.37–2.37)	1.57^a^ (1.08–2.28)	1.75^b,c^ (1.18–2.59)	0.73 (.32–1.67)	0.18 (.03–1.23)
GI.5	1.47 (.80–2.71)	**0.89 (.31–2.51)**	0.89 (.44–1.82)	0.67 (.22–2.07)	1.17 (.76–1.80)	0.81 (.42–1.57)	1.29 (.66–2.53)	1.30 (.42–4.07)
GI.7	1.17 (.72–1.90)	0.90 (.44–1.81)	**0.19** ^**a**^ **(.04–.79)**	0.85 (.33–2.33)	1.10 (.76–1.60)	1.06 (.71–1.57)	0.94 (.44–2.00)	1.09 (.48–2.48)
GII.2	1.36 (.79–2.33)	0.27 (.07–1.07)	0.53 (.21–1.29)	**0.74 (.30–1.86)**	1.16 (.78–1.72)	1.33 (.88–2.01)	3.42^b,c^ (2.00–5.82)	1.05 (.44–2.51)
GII.4	0.52^b,c^ (.33–.82)	1.03 (.52–2.03)	0.74 (.44–1.24)	0.41^a^ (.20–.84)	**0.39** ^**b,c**^ **(.27–.54)**	0.91 (.66–1.27)	0.79 (.43–1.45)	1.13 (.67–1.92)
GII.6	1.09 (.71–1.70)	1.18 (.64–2.18)	0.73 (.43–1.26)	1.61 (.84–3.09)	0.96 (.70–1.33)	**0.52** ^**b,c**^ **(.32–.82)**	1.73^a^ (1.03–2.91)	2.19^a^ (1.19–4.03)
GII.17	2.06^b,c^ (1.29–3.30)	0.61 (.17–1.23)	0.77 (.39–1.52)	0.94 (.39–1.26)	0.71 (.43–1.17)	1.06 (.69–1.61)	**0.38 (.12–1.20)**	0.91 (.32–2.58)
GII.23	0.76 (.41–1.41)	1.22 (.61–2.44)	0.72 (.32–1.62)	0.43 (.14–1.29)	0.83 (.54–1.27)	1.03 (.72–1.47)	0.90 (.42–1.95)	**1.14 (.49–2.65)**

Hazard ratios of being infected with the same genotype (highlighted with bold text) or a different genotype among children with vs without prior infection with genotypes are listed in the left-hand column. Values between 0 and 1 denote reduced risk of subsequent infection among the previously exposed; values > 1 denote increased risk of subsequent infection among the previously exposed. Hazard ratios included adjustment for length-for-age *z* score and the duration of exclusive breastfeeding.

Abbreviation: CI, confidence interval.

^a^ *P* < .05 (significantly different from the null hypothesis that there is no relationship between prior and future infection).

^b^ *P* < .01 (strong significant finding).

^c^Holds after Holm correction (*P* < .007).

Notably, children with prior GI.3 exposure had a 75% increased hazard of GII.6 infection (HR, 1.75; *P* = .005) and a tendency of a 57% increased hazard of subsequent GII.4 infections (HR, 1.57; *P* = .018) that failed to retain significance after correction for multiple comparisons. Similarly, prior GII.2 exposure was associated with a >3-fold higher subsequent incidence of GII.17 infection (HR, 3.42; *P* < .001), and prior GII.17 exposure was with increased subsequent GI.3 infections (HR, 2.06; *P* = .003). Prior GII.6 exposure trended toward an association with an augmentation in subsequent risk of infection of 73% to GII.17 (HR, 1.73; *P* = .037) and a >2-fold increase in risk of infection with GII.23 (HR, 2.19; *P* = .011); however, these associations failed to meet criteria for statistical significance. Sensitivity analysis was also done with time to new infection windows of 14 and 90 days, which did not significantly alter results (Supplementary Table 1). To determine the likelihood of impact of multiple comparisons on our findings, an estimated 3.2 rejections of the null hypothesis could be anticipated by chance in this set of comparisons, whereas 12 were observed; thus, the probability of a global null hypothesis in regard to history of norovirus infections not altering the subsequent risk of infection is *P* < .0001. To further confirm our observations, we also ran the model on *Campylobacter* data from the parent study [12] and found that there was no evidence of altered incidence of norovirus genotypes following infection (Supplementary Table 2), supporting that the observed associations we saw in norovirus may have biologic underpinnings.

We replicated the analyses on the impact of prior norovirus infections on the occurrence of subsequent symptomatic illness with 2 genotypes, GII.4 and GII.6, which had sufficient frequency for modeling of clinical episodes (Table 4), and demonstrated that the degree of protection against symptomatic infection was nearly identical to all (symptomatic and asymptomatic) GII.4 and GII.6 infections.

**Table 4.** Adjusted Hazard Ratios for the Risk of Symptomatic Norovirus Diarrhea Caused by GII.4 and GII.6, After Prior Infection With 1 of 8 Genotypes, Among 194 Children With Complete Surveillance Data From Birth Until 24 Months of Age

Genotype of Prior Infection	Adjusted HR (95% CI) for Symptomatic Infection	
	GII.4 (n = 92)	GII.6 (n = 51)
GI.3	1.43 (.85–2.40)	1.12 (.50–2.51)
GI.5	0.76 (.30–1.89)	0.78 (.27–2.25)
GI.7	1.19 (.68–2.10)	1.45 (.80–2.61)
GII.2	1.32 (.75–2.30)	1.07 (.48–2.40)
GII.4	0.29^a^ (.18–.48)	1.06 (.59–1.90)
GII.6	1.05 (.65–1.66)	0.70 (.38–1.27)
GII.17	0.76 (.40–1.41)	1.21 (.66–2.21)
GII.23	0.72 (.35–1.48)	0.99 (.52–1.88)

HRs include adjustment for length-for-age *z* score and the duration of exclusive breastfeeding.

Abbreviations: CI, confidence interval; HR, hazard ratio.

^a^Holds after Holm correction (*P* < .007).

## DISCUSSION

This study is the first to provide estimates of homotypic and heterotypic protection following naturally acquired norovirus infection in a Peruvian birth cohort. This cohort had a high incidence of norovirus disease and provided an excellent opportunity to follow natural infections in children from birth to 2 years of age. Norovirus genotypes GI.3, GII.4, and GII.6 were associated with 54% of all infections, which is similar to the major genotypes in pediatric populations globally [23, 24]. We found >60% homotypic protection to the most frequently detected norovirus genotype GII.4 and heterotypic protection of 48% against GI.3, the third most prevalent genotype in this large study. Given the high prevalence of these infections, a GII.4 component has the potential to avert a large number of episodes of norovirus gastroenteritis.

In this cohort, 87% of the children experienced at least 1 (range, 1–17) norovirus infection and in addition to GI.3, GII.4, and GII.6 viruses, a wide range of genetically diverse norovirus genotypes co-circulated, including all known GI and almost all GII genotypes. High prevalence of norovirus in young children with relatively low prevalence in healthy controls has been described consistently in several Latin American countries [8, 11, 25–29], and all children in this cohort are secretor positive [29]. However, asymptomatic infections and prolonged virus shedding were common in this study population, which is consistent with reports from other birth cohort studies [20, 26, 30, 31]. Interestingly, we detected GII.4 Sydney viruses in samples that were collected in 2010, 2 years before this pandemic strain was first reported [32] This has been reported by others [33, 34] and in 1 of these reports, GII.4 Sydney strains were detected in stool samples from sporadic norovirus cases as early as 1994 [33], highlighting the importance of including typing of norovirus trains from sporadic cases of gastroenteritis in surveillance programs for early detection of emerging strains with pandemic potential.

We employed Kaplan-Meier and Cox proportional hazards regression models to provide robust estimates of homotypic and heterotypic protection following natural infections. Although typical duration of norovirus shedding is days to weeks [20], in young infants with an immature immune system, virus can sometimes be shed for months or longer [30]. We defined our cutoff for persistent infections to 30 days as suggested in a recent review of the literature on the effect of long-term shedding on norovirus risk [20]. Sensitivity analysis with the cutoff for persistent infections set at 14 and 90 days did not significantly alter the results.

Several previous studies have shown that children can be reinfected with norovirus multiple times during the first 5 years of life and that the majority of these reinfections occur with different norovirus genotypes or different GII.4 variants [5, 8, 9, 27, 35, 36]. However, due to study design, sample size, and/or methodology limitations, none of these studies performed survival analysis to estimate the level of protection against individual genotypes, which is key information required to assess the feasibility and composition of a norovirus vaccine in pediatric populations. The successful typing of a large number of the norovirus infections in this study allowed for estimates of homotypic and heterotypic protection and probabilities of reinfection following initial norovirus infection. While GII.4 showed both homotypic and heterotypic protection, GII.6 also exhibited homotypic protection of a 48% reduction in risk following a documented infection.

A notable finding of our study was the increased vulnerability seen to certain norovirus infections following infections with a different genotype, especially those involving GI.3, GII.6, and GII.17 strains. GII.17 infections enhanced the susceptibility to GI.3 infections by 2-fold. An augmentation of risk of infections with GII.17 strains following GII.2 and GII.6 infections was also observed. To our knowledge, this is the first time this phenomenon has been described for an enteropathogen other than bocavirus [36]. This is the first report of 1 norovirus infection positively influencing the subsequent risk of acquisition of other noroviruses, which merits further investigation. Biological plausibility of this observation is found in several recent studies of the repertoire of serological responses in the murine model [37]. Lindesmith et al recently profiled the antibody repertoire following immunization in humans and found that the immunoglobulin G response to norovirus vaccine is dominated by the boosting of a limited number of clonotypes. It is also notable that murine and bovine noroviruses have exhibited dual tropism, infecting not only epithelial cells, but dendritic cells and cells with monocyte morphologies, raising the possibility of antibody dependent enhancement-like mechanisms [37]. We further evaluated whether *Campylobacter* infection altered the subsequent risk of norovirus infection, but no effect was noted, suggesting that confounding was not a likely explanation underlying these findings. Clearly, these findings warrant further investigation as multicomponent norovirus vaccines are under development and enhancement has been reported even across genogroups [38].

Our study has several limitations. First, although we were able to detect coinfections of GI and GII noroviruses in 4.1% of the participants, the number of coinfections is likely higher because conventional RT-PCR assays used for genotyping are unable to detect coinfections with viruses of the same genogroup. Use of next-generation sequencing technology would assist in providing a more accurate picture of the number of mixed infections, highlighting the increased risk for recombination between different norovirus strain to occur in this population. Second, the age distribution of infections might be affected by the fact that routine sampling was performed monthly in year 1 but quarterly in year 2. Hence, some infections in the second year might be missed, leading to underestimated overall cumulative incidence that could skew the distribution to year 1 of the participants. The 30-day cutoff was previously suggested for studies done in controlled experiments using only healthy individuals [20] and thus might not represent the accurate cutoff for considering persistent infections in natural infections in malnourished children. The increase hazard of reinfection with different genotypes does not necessarily represent an increase in the severity of disease, as both symptomatic and asymptomatic infections were included.

In conclusion, children in this Peruvian birth cohort showed homotypic and heterotypic protection to GII.4 noroviruses, which supports the feasibility of current norovirus vaccine formulations that include GII.4. However, enhanced susceptibility observed following infection with several other genotypes merits further studies prior to inclusion of additional genotypes in a multivalent vaccine in communities with high norovirus diversity.

## Supplementary Data

Supplementary materials are available at *Clinical Infectious Diseases* online. Consisting of data provided by the authors to benefit the reader, the posted materials are not copyedited and are the sole responsibility of the authors, so questions or comments should be addressed to the corresponding author.

ciaa019_suppl_Supplementary_Tables

## Notes

***Author contributions.*** J. V., P. C., and M. K. designed the study, interpreted the data, and wrote the manuscript. H. B. and P. C. tested and genotyped all stool samples. S. R. performed the hazard analyses. L. H. M. helped confirm the statistical analyses. P. P. Y., M. S. S., and M. P. O. critically reviewed the manuscript and read and approved the final version.

***Acknowledgments.*** The authors thank the Etiology, Risk Factors and Interactions of Enteric Infections and Malnutrition and the Consequences for Child Health and Development (MAL-ED) study participants and their children for their collaboration, and Peter Teunis for critical review of the manuscript.

***Financial support.*** The MAL-ED project was carried out as a collaborative project supported by the Bill & Melinda Gates Foundation, the Foundation for the National Institutes of Health (NIH), and the NIH Fogarty International Center. Additional funding was provided by the Bill & Melinda Gates Foundation (grant number OPP1066146 to M. N. K.); the Fisher Center for Environmental Infectious Diseases at the Johns Hopkins School of Medicine; and the intramural food safety program at the CDC (to P. C. and J. V.).

***Potential conflicts of interest.*** The authors: No reported conflicts of interest. All authors have submitted the ICMJE Form for Disclosure of Potential Conflicts of Interest.

## References

[1] Ahmed SM, Hall AJ, Robinson AE, et al. Global prevalence of norovirus in cases of gastroenteritis: a systematic review and meta-analysis. Lancet Infect Dis 2014; 14:725–30. 24981041 10.1016/S1473-3099(14)70767-4 PMC8006533

[2] Pires SM, Fischer-Walker CL, Lanata CF, et al. Aetiology-specific estimates of the global and regional incidence and mortality of diarrhoeal diseases commonly transmitted through food. PLoS One 2015; 10:e0142927. 26632843 10.1371/journal.pone.0142927 PMC4668836

[3] Green K Caliciviridae: the noroviruses. In: Knipe DM, Howley PM, eds. Fields virology. Philadelphia, PA: Lippincott Williams & Wilkins, 2013.

[4] Chhabra P, de Graaf M, Parra GI, et al. Updated classification of norovirus genogroups and genotypes. J Gen Virol 2019; 100:1393–406. 31483239 10.1099/jgv.0.001318 PMC7011714

[5] Parra GI, Squires RB, Karangwa CK, et al. Static and evolving norovirus genotypes: implications for epidemiology and immunity. PLoS Pathog 2017; 13:e1006136. 28103318 10.1371/journal.ppat.1006136 PMC5283768

[6] Teunis PF, Sukhrie FH, Vennema H, Bogerman J, Beersma MF, Koopmans MP Shedding of norovirus in symptomatic and asymptomatic infections. Epidemiol Infect 2015; 143:1710–7. 25336060 10.1017/S095026881400274X PMC9507237

[7] Wyatt RG, Dolin R, Blacklow NR, et al. Comparison of three agents of acute infectious nonbacterial gastroenteritis by cross-challenge in volunteers. J Infect Dis 1974; 129:709–14. 4209723 10.1093/infdis/129.6.709

[8] Saito M, Goel-Apaza S, Espetia S, et al; Norovirus Working Group in Peru Multiple norovirus infections in a birth cohort in a Peruvian periurban community. Clin Infect Dis 2014; 58:483–91. 24300042 10.1093/cid/cit763 PMC3905757

[9] Cannon JL, Lopman BA, Payne DC, Vinjé J Birth cohort studies assessing norovirus infection and immunity in young children: a review. Clin Infect Dis 2019; 69:357–65. 30753367 10.1093/cid/ciy985 PMC7962893

[10] Glass RI, Parashar UD, Estes MK Norovirus gastroenteritis. N Engl J Med 2009; 361:1776–85. 19864676 10.1056/NEJMra0804575 PMC3880795

[11] Rouhani S, Peñataro Yori P, Paredes Olortegui M, et al; Etiology, Risk Factors, and Interactions of Enteric Infections and Malnutrition and the Consequences for Child Health and Development Project (MAL-ED) Network Investigators Norovirus infection and acquired immunity in 8 countries: results from the MAL-ED study. Clin Infect Dis 2016; 62:1210–7. 27013692 10.1093/cid/ciw072 PMC4845786

[12] MAL-ED Network. The MAL-ED study: a multinational and multidisciplinary approach to understand the relationship between enteric pathogens, malnutrition, gut physiology, physical growth, cognitive development, and immune responses in infants and children up to 2 years of age in resource-poor environments. Clin Infect Dis 2014; 59:S193–206. 25305287 10.1093/cid/ciu653

[13] Yori PP, Lee G, Olórtegui MP, et al. Santa Clara de Nanay: the MAL-ED cohort in Peru. Clin Infect Dis 2014; 59:S310–6. 25305303 10.1093/cid/ciu460

[14] Houpt E, Gratz J, Kosek M, et al; MAL-ED Network Investigators Microbiologic methods utilized in the MAL-ED cohort study. Clin Infect Dis 2014; 59:S225–32. 25305291 10.1093/cid/ciu413 PMC4204609

[15] Cannon JL, Barclay L, Collins NR, et al. Genetic and epidemiologic trends of norovirus outbreaks in the United States from 2013 to 2016 demonstrated emergence of novel GII.4 recombinant viruses. J Clin Microbiol 2017; 55:2208–21. 28490488 10.1128/JCM.00455-17 PMC5483924

[16] Kroneman A, Vennema H, Deforche K, et al. An automated genotyping tool for enteroviruses and noroviruses. J Clin Virol 2011; 51:121–5. 21514213 10.1016/j.jcv.2011.03.006

[17] Kumar S, Stecher G, Li M, Knyaz C, Tamura K MEGA X: molecular evolutionary genetics analysis across computing platforms. Mol Biol Evol 2018; 35:1547–9. 29722887 10.1093/molbev/msy096 PMC5967553

[18] Prentice RL, Kalbfleisch JD Hazard rate models with covariates. Biometrics 1979; 35:25–39. 497336

[19] Raboud J, Breslow NE Efficiency gains from the addition of controls to matched sets in cohort studies. Stat Med 1989; 8:977–85. 2799126 10.1002/sim.4780080808

[20] Milbrath MO, Spicknall IH, Zelner JL, Moe CL, Eisenberg JN Heterogeneity in norovirus shedding duration affects community risk. Epidemiol Infect 2013; 141:1572–84. 23507473 10.1017/S0950268813000496 PMC9155277

[21] Holm S A simple sequentially rejective multiple test procedure. Scand J Stat 1979; 6:65–70.

[22] Chhabra P, Aswath K, Collins N, et al. Near-complete genome sequences of several new norovirus genogroup II genotypes. Genome Announc 2018; 6:e00007-18. 29439030 10.1128/genomeA.00007-18 PMC5805868

[23] Hassan F, Kanwar N, Harrison CJ, et al. Viral etiology of acute gastroenteritis in <2-year-old US children in the post-rotavirus vaccine era. J Pediatric Infect Dis Soc 2019; 8:414–21. 30184153 10.1093/jpids/piy077

[24] Mans J Norovirus infections and disease in lower-middle and low-income countries, 1997–2018. Viruses 2019; 11:E341. 30974898 10.3390/v11040341 PMC6521228

[25] Becker-Dreps S, Bucardo F, Vilchez S, et al. Etiology of childhood diarrhea after rotavirus vaccine introduction: a prospective, population-based study in Nicaragua. Pediatr Infect Dis J 2014; 33:1156–63. 24879131 10.1097/INF.0000000000000427 PMC4216626

[26] Lopman BA, Trivedi T, Vicuña Y, et al. Norovirus infection and disease in an Ecuadorian birth cohort: association of certain norovirus genotypes with host FUT2 secretor status. J Infect Dis 2015; 211:1813–21. 25505295 10.1093/infdis/jiu672 PMC4425937

[27] Nelson MI, Mahfuz M, Chhabra P, et al. Genetic diversity of noroviruses circulating in a pediatric cohort in Bangladesh. J Infect Dis 2018; 218:1937–42. 30053045 10.1093/infdis/jiy454 PMC6217719

[28] O’Ryan ML, Lucero Y, Prado V, et al. Symptomatic and asymptomatic rotavirus and norovirus infections during infancy in a Chilean birth cohort. Pediatr Infect Dis J 2009; 28:879–84. 19672213 10.1097/INF.0b013e3181a4bb60

[29] Yori PP, Schwab K, Gilman RH, et al. Norovirus highly prevalent cause of endemic acute diarrhea in children in the Peruvian Amazon. Pediatr Infect Dis J 2009; 28:844–7. 19636281 10.1097/INF.0b013e3181a24730

[30] Murata T, Katsushima N, Mizuta K, Muraki Y, Hongo S, Matsuzaki Y Prolonged norovirus shedding in infants <or=6 months of age with gastroenteritis. Pediatr Infect Dis J 2007; 26:46–9. 17195705 10.1097/01.inf.0000247102.04997.e0

[31] Kirkwood CD, Streitberg R Calicivirus shedding in children after recovery from diarrhoeal disease. J Clin Virol 2008; 43:346–8. 18789755 10.1016/j.jcv.2008.08.001

[32] Eden JS, Tanaka MM, Boni MF, Rawlinson WD, White PA Recombination within the pandemic norovirus GII.4 lineage. J Virol 2013; 87:6270–82. 23536665 10.1128/JVI.03464-12 PMC3648122

[33] Allen DJ, Trainor E, Callaghan A, O’Brien SJ, Cunliffe NA, Iturriza-Gómara M Early detection of epidemic GII-4 norovirus strains in UK and Malawi: role of surveillance of sporadic acute gastroenteritis in anticipating global epidemics. PLoS One 2016; 11:e0146972. 27115152 10.1371/journal.pone.0146972 PMC4846118

[34] Mugyia AE, Ndze VN, Akoachere JTK, et al. Molecular epidemiology of noroviruses in children under 5 years of age with acute gastroenteritis in Yaounde, Cameroon. J Med Virol 2018; 91:738–43. 10.1002/jmv.25380 PMC8978256 30570784

[35] Nelson MI, Mahfuz M, Chhabra P, et al. Genetic diversity of noroviruses circulating in a pediatric cohort in Bangladesh. J Infect Dis 2018; 218:1937–42. 30053045 10.1093/infdis/jiy454 PMC6217719

[36] Sakon N, Yamazaki K, Nakata K, et al. Impact of genotype-specific herd immunity on the circulatory dynamism of norovirus: a 10-year longitudinal study of viral acute gastroenteritis. J Infect Dis 2015; 211:879–88. 25210139 10.1093/infdis/jiu496

[37] Wobus CE The dual tropism of noroviruses. J Virol 2018; 92:e01010-17. 10.1128/JVI.01010-17 PMC6069179 29848591

[38] Lindesmith LC, Ferris MT, Mullan CW, et al. Broad blockade antibody responses in human volunteers after immunization with a multivalent norovirus VLP candidate vaccine: immunological analyses from a phase I clinical trial. PLoS Med 2015; 12:e1001807. 25803642 10.1371/journal.pmed.1001807 PMC4371888

