# Supplementary material for: Homotypic and Heterotypic Protection and Risk of Reinfection Following Natural Norovirus Infection in a Highly Endemic Setting
Source: Clin Infect Dis. 2020 Jan 9;72(2):222–9. doi: 10.1093/cid/ciaa019 (PMC7840104; doi:10.1093/cid/ciaa019)
Supplement: ciaa019_suppl_Supplementary_Tables [file ciaa019_suppl_supplementary_tables.docx]

Supplementary S1. Adjusted hazard ratios denoting homotypic and heterotypic protection or cross-reactivity between norovirus genotypes among 194 children in Peru if the time delineating a new infection between detections is expanded from 30 days to 90 days

|  | **Adjusted Hazard Ratio [95% Confidence Interval]** | | | | | | | |
| --- | --- | --- | --- | --- | --- | --- | --- | --- |
| **Prior infection** | GI.3  (n=71) | GI.5  (n=48) | GI.7  (n=58) | GII.2 (n=44) | GII.4 (n=182) | GII.6 (n=141) | GII.17 (n=53) | GII.23 (n=45) |
| GI.3 | 0.23*  [0.11, 0.74] | 0.67  [0.29, 1.54] | 0.60  [0.27, 1.36] | 0.93  [0.35, 2.45] | 1.43  [0.99, 2.05] | 1.79**^  [1.22, 2.63] | 0.74  [0.32, 1.67] | 0.19  [0.03, 1.33] |
| GI.5 | 1.51  [0.82, 2.79] | 0.52  [0.17, 1.57] | 0.90  [0.44, 1.85] | 0.70  [0.23, 2.15] | 1.18  [0.79, 1.78] | 0.89  [0.44, 1.67] | 1.29  [0.65, 2.53] | 1.41  [0.45, 4.42] |
| GI.7 | 1.20  [0.74, 1.94] | 0.95  [0.48, 1.91] | 0.09*  [0.01, 0.66] | 0.88  [0.33, 2.31] | 1.08  [0.75,1.55] | 1.05  [0.71, 1.56] | 0.94  [0.44, 1.99] | 1.21  [0.54, 2.72] |
| GII.2 | 1.40  [0.82, 2.40] | 0.28  [0.07, 1.11] | 0.54  [0.22, 1.32] | 0.55  [0.19, 1.60] | 1.05  [0.71, 1.55] | 1.24  [081, 1.91] | 3.42**^  [2.01, 5.81] | 1.12  [0.47, 2.66] |
| GII.4 | 0.55**  [0.35, 0.85] | 1.06  0.55, 2.05] | 0.77  [0.46, 1.30] | 0.42*  [0.20, 0.88] | 0.27**^  [0.18, 0.39] | 0.98  [0.70, 1.36] | 0.79  [0.43, 1.45] | 1.08  [0.63, 1.83] |
| GII.6 | 1.03  [0.66, 1.59] | 1.22  [0.67, 2.22] | 0.77  [).45, 1.33] | 1.77  [0.95, 3.33] | 1.03  [0.74, 1.44] | 0.36**^  [0.22, 0.59] | 1.79*  [1.07, 3.00] | 2.10*  [1.13, 3.91] |
| GII.17 | 1.98**^  [1.23, 3.19] | 0.50  [0.15, 1.64] | 0.79  [0.40, 1.56] | 0.99  [0.41, 2.36] | 0.71  {0.44, 1.16] | 1.05  [0.70, 1.58] | 0.39  [0.12, 1.22] | 0.98  [0.34, 2.77] |
| GII.23 | 0.77  [0.42, 1.42] | 1.31  [0.66, 2.60] | 0.74  [0.33, 1.66] | 0.44  [0.15, 1.34] | 0.90  [0.59, 1.39] | 1.08  [0.76, 1.54] | 0.90  [0.41, 1.95] | 0.64  [0.22, 1.85] |

** p<0.05 (significantly different from the null hypothesis that there is no relationship between prior and future infection)*

*** p<0.01 (strong significant finding)*

***^ Holds after Holm’s correction; p<0.007*

Table S2. Adjusted hazard ratios for subsequent risk of infection with specific genotypes of norovirus, after prior Campylobacter infection among 194 children.

| **Subsequent infections** | **HR [95%CI]** |
| --- | --- |
| **All infections** |  |
| GI.3 (n=73) | 1.42 [0.59, 3.42] |
| GI.5 (n=51) | 1.35 [0.56, 3.27] |
| GI.7 (n=58) | 0.81 [0.40, 1.66] |
| GII.2 (n=45) | 0.82 [0.38, 1.74] |
| GII.4 (n=195) | 0.74 [0.53, 1.04] |
| GII.6 (n=150) | 1.00 [0.64, 1.54] |
| GII.17 (n=53) | 0.79 [0.40, 1.57] |
| GII.23 (n=49) | 0.95 [0.40, 2.23] |
| **Symptomatic infections** |  |
| GII.4 (n=89) | 0.89 [0.56 1.43] |
| GII.6 (n=62) | 1.38 [0.79, 2.39] |
